# The cumulative live birth rate and cost-effectiveness of the clomiphene and gonadotropin cotreatment protocol versus the mid-luteal GnRH agonist protocol in women over 35 years old

**DOI:** 10.1038/s41598-024-63842-x

**Published:** 2024-06-05

**Authors:** Yanhui Li, Dan Luo, Tao Li, Hui Ding, Yi Liu

**Affiliations:** Department of Obstetrics and Gynecology, Union Hospital, Tongji Medical College, Huazhong University of Science and Technology, Wuhan, 430022 Hubei China

**Keywords:** Infertility, Outcomes research, Reproductive techniques

## Abstract

The decrease in assisted reproductive technology success among older women, attributed to decreased oocyte quantity and quality, poses a significant challenge. Currently, no consensus on the optimal ovarian stimulation protocol for older women undergoing IVF exists. This retrospectively registered cohort study aimed to compare the cumulative live birth rate (CLBR), time to live birth (TTLB), and cost-effectiveness among women older than 35 years who were receiving either the gonadotropin-releasing hormone agonist (GnRHa) or clomiphene citrate and gonadotropin cotreatment with ovarian stimulation (CC cotreatment) protocol. To compare treatment outcomes, we performed propensity score matching (PSM) on 2871 IVF cycles in women older than 35 years who received either the GnRHa or CC cotreatment protocol, resulting in 375 cycles in each group. Additionally, a decision tree model was utilized to assess the cost-effectiveness of the two protocols. Following PSM, both groups had similar baseline characteristics. The CC cotreatment protocol resulted in a greater rate of cycle cancellation (13.07% vs. 8.00%, *p* = 0.032), but the groups maintained comparable fertilization rates and embryo quality. Although the TTLB was longer in the CC cotreatment group, the CLBR per initial cycle (41.07% vs. 45.33%, *p* = 0.269) and delivery outcomes were similar between the two groups at the 24 months follow-up. Additionally, the average cost per live birth in the CC cotreatment group was 21.27% lower than in the GnRHa group (¥32,301.42 vs. ¥39,174.22). In conclusion, for women older than 35 years undergoing IVF, the CC cotreatment protocol offered a comparable CLBR to the GnRHa protocol but with reduced costs, indicating its potential as a viable and cost-effective ovarian stimulation option.

*Clinical trial registration*: https://www.chictr.org.cn/, identifier [ChiCTR2300076537].

## Introduction

According to epidemiological data, the incidence of infertility is increasing in many countries and regions around the world^1^, including in China^2^. Increased chances for education and career advancement, as well as the promotion of effective contraceptive measures, are causing an increasing number of women to choose to have children after the age of 35 years, which is a major factor contributing to the increase in the incidence of infertility^3^. Age-related fertility decline^4^ accounts for an increasing proportion of the causes of assisted reproductive technology. However, the pregnancy rate after IVF treatment in women older than 35 years of age is also significantly reduced due to a decrease in oocyte quantity and quality^5,6^ and probable changes in endometrial receptivity^7^. The current primary strategy to compensate for age-related decreases in oocyte quality and quantity is to maximize the ovarian response by developing patient-specific protocols^8^. Therefore, various ovarian stimulation protocols, such as the gonadotrophin releasing hormone (GnRH) agonist protocol^9^, GnRH antagonist (GnRHA) protocol, mild-stimulation protocol^10^, progestin-primed ovarian stimulation protocol, double stimulation/dual stimulation protocol, and modified natural cycle protocol^11^, have been used for older women^12^, but the advantages and disadvantages of each protocol in terms of efficacy, safety, and cost-effectiveness still need to be further clarified.

Clomiphene citrate (CC) is a selective estrogen receptor modulator with strong anti-estrogen effects and weak estrogen effects. CC is orally administered, inexpensive and clinically accessible. The pharmacological mechanism of clomiphene is still unclear. Currently, it is believed that low-dose drugs may act on the hypothalamus, compete with estrogen to bind to receptors, block estrogen negative feedback, and promote the release of GnRH and endogenous gonadotropin^13^. CC was first approved by the United States Food and Drug Administration for human ovulation stimulation in 1961; its use is believed to improve ovarian responsiveness and minimize the gonadotropin dosage by promoting the release of endogenous gonadotropin. The 2020 European Society of Human Reproduction and Embryology (ESHRE) guidelines also recommend the use of clomiphene alone or in combination with gonadotropin for ovarian stimulation in patients predicted to have a poor response^14^. Currently, the most commonly discussed ovarian stimulation protocol in combination with clomiphene and gonadotropin is the mild-stimulation protocol. Across high responders, normal responders, and poor responders, numerous studies have indicated that while the combination of gonadotropin and clomiphene can lower the cost of ovarian stimulation without compromising embryo quality, it also leads to an increase in the cycle cancellation rate, a decrease in the number of oocytes retrieved, and a decrease in the cumulative pregnancy rate^15–18^. As a result, the mild stimulation protocol has a lower application efficiency in individuals with a “nonseverely declining” ovarian reserve than do the conventional GnRH agonist (GnRHa) or GnRHA protocols^19^. Recent research has also attempted to extend the usage of CC until the trigger day. Because the antiestrogenic effect of CC can inhibit endogenous LH peaks, it can reduce the need for GnRHa or GnRHA, decreasing the cost of ovarian stimulation^20,21^. Based on this consideration, our center has used a continuous combination of clomiphene and an adequate dose of gonadotrophin ovarian stimulation (we named this combination the clomiphene citrate cotreatment protocol, (CC cotreatment) protocol) in older women and has achieved good results. In this study, we retrospectively analyzed the cumulative live birth rate (CLBR), time to live birth (TTLB) and cost-effectiveness ratio (C/E) of the CC cotreatment and GnRHa protocols in women aged > 35 years to evaluate the application value of the CC cotreatment protocol in older women.

## Materials and methods

### Study design

This was a retrospectively registered cohort study. All patients who received IVF/ICSI treatment at the Reproductive Center of Union Hospital, Tongji Medical College, and Huazhong University of Science and Technology between January 2016 and June 2021 were included. Patients who met the following criteria were included in the statistical analysis: female patients > 35 years old and < 50 years old; patients with indications for IVF or ICSI treatment; and patients who underwent ovarian stimulation using either the GnRHa protocol or the CC cotreatment protocol (for comprehensive implementation methods of these protocols, see Supplementary Fig. S1). Patients who met any of the following criteria were excluded: hysterosalpingography or hysteroscopy indicating uterine abnormalities, recurrent miscarriage, rAFS stage III–IV (rAFS stage) endometriosis, donor oocyte/sperm cycles, preimplantation genetic testing cycles, or oocyte freezing cycles. All patients who became pregnant after IVF/ICSI treatment were followed up regularly during pregnancy, and postbirth follow-up was performed within one week after delivery. The decision to use ovarian stimulation with either the CC cotreatment or GnRHa protocol was jointly made by the patient's attending physician and the patient herself, with the input of her partner, after obtaining full informed consent. Our center did not have any mandatory requirements for the ovarian stimulation protocol for women older than 35 years during the observation period of this study. The two groups were further matched using baseline data for standard propensity score matching (PSM).

### Fertilization, embryo evaluation and embryo transfer in the CC cotreatment group

For the CC cotreatment group, oocyte fertilization was achieved by IVF or ICSI based on the male partner’s sperm quality. For IVF, approximately 20,000 motile sperm were added to each oocyte. ICSI was conducted if the total motile sperm concentration was less than 5 × 10^6^/ml or if the proportion of normal sperm was less than 1%. The presence of two pronuclei (2PN) 16–18 h after insemination was regarded as normal fertilization. The embryos were evaluated after 72 h of in vitro culture based on blastomere homogeneity and the embryo fragmentation rate^22^. Embryos with 6–8 cells and a grade of 1 or 2 were regarded as top-quality embryos. Embryos in the CC cotreatment group were cultured in G2-plus medium (Vitrolife, Gothenburg, Sweden) for an additional 2–4 days for blastocyst development. Blastocysts were graded using Gardner’s grading scale^23^. All patients in the CC cotreatment group underwent frozen embryo transfer using the “freeze-all” strategy.

Most frozen embryo transfer (FET) cycles involved hormone replacement cycles. The specifics of the vitrification, warming technique and endometrial preparation were previously described^24^. A maximum of two cleavage-stage embryos or blastocysts were transferred each time under the guidance of abdominal ultrasound. First, 10 mg of twice-daily oral dydrogesterone (Duphaston, Abbott, USA) and 90 mg of vaginal progesterone gel (Crinone; Merck Serono) were used for luteal support. Luteal support was initiated on the day of endometrial transformation. At 12–14 days after embryo transfer, pregnancy tests were conducted. For pregnant patients, luteal support continued until 12 weeks of gestation.

### Fertilization, embryo evaluation and transfer in the GnRHa group

Patients in the GnRHa group underwent the same procedures for in vitro fertilization, embryo culture, and evaluation as those in the CC cotreatment group. However, eligible patients in the GnRHa group underwent fresh embryo transfer on Day 3, Day 5, or Day 6. Similarly, a maximum of two embryos at either the cleavage or blastocyst stage were transferred each time. Luteal support for patients who underwent fresh embryo transfer began on the second day following oocyte retrieval and continued until the 12th week of pregnancy. Blastocyst culture was performed for patients who did not undergo fresh embryo transfer and had surplus embryos after fresh embryo transfer. For patients who underwent FET in the GnRHa group, the protocols for endometrial preparation and luteal support were identical to those used in the CC cotreatment group.

### Outcome measures

The primary outcome measures of this study were the CLBR within 24 months after the initiation of ovarian stimulation and the TTLB. The secondary outcome measures included the incidence of premature LH surge, fertilization rate, cycle cancellation rate, clinical pregnancy rate and abortion rate. Furthermore, the number of retrieved oocytes, the percentage of mature oocytes, the number of available embryos, the incidence of ovarian hyperstimulation syndrome (OHSS), the percentage of multiple pregnancies, birth weight, and gestational age were compared between the two groups.

A premature LH surge was defined as a serum LH level ≥ 10 mIU/mL or an increase of more than 2.5 times above the basal level during ovarian stimulation^25,26^. An available embryo on Day 3 was defined as an embryo that had a minimum of 4 blastomeres, had a fragmentation rate below 25%, and had undergone cleavage within the previous 24 h period^27^. A live birth was defined as the birth of a live baby at 28 weeks of gestation or with a birth weight greater than 1000 g. The CLBR was defined as the number of cycles resulting in at least one live birth among all fresh embryo or frozen embryo transfer cycles divided by the total number of initiated cycles over a 2 years period. A clinical pregnancy was defined as the identification of a gestational sac 4 weeks after embryo transfer. The miscarriage rate was defined as the number of miscarriages before 28 weeks of gestation divided by the total number of clinical pregnancies. The cycle cancellation rate was calculated as the number of patients with no available embryos divided by the total number of patients for whom ovarian stimulation was initiated. The time to live birth was the interval between the initiation of ovarian stimulation and live birth. When multiple live births occurred within a single ovarian stimulation cycle, only the first live birth was included.

### Cost-effectiveness analysis

**Figure 1:**
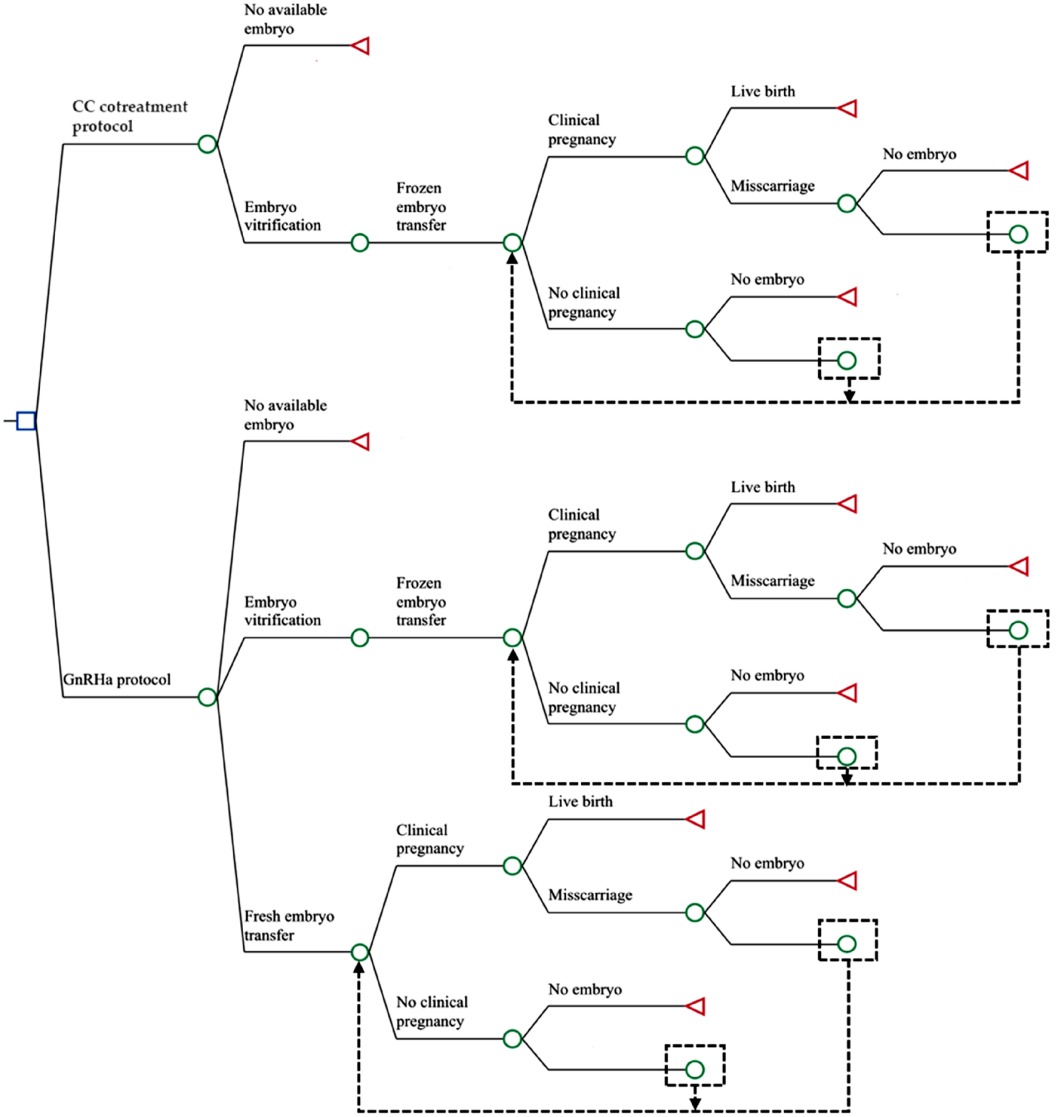
Decision tree model based on the real-world data of this study. *Note* CC cotreatment, clomiphene citrate and gonadotropin cotreatment; GnRHa, gonadotropin-releasing hormone agonist; nodes within the model are marked by circles, and triangles define endpoints.

The decision tree model TreeAge Pro 2019 (TreeAge software, Inc., Williamstown, MA, USA) was used for cost-effectiveness analysis of the two ovarian stimulation protocols (Fig. 1). A decision tree is a decision-making method that simulates a group of patients making decisions according to a predefined method, with associated probabilities, costs, and outcomes. Cost-effectiveness analysis based on a decision tree model is a useful method for assessing the costs and outcomes of newly adopted interventions^28^.

The decision tree model constructed in this study incorporated key and costly major clinical events, including pre-IVF examination, ovarian stimulation, oocyte retrieval, in vitro embryo culture, embryo freezing and warming, embryo transfer, luteal support, miscarriage and childbirth. For both groups of patients, each embryo transfer had three possible outcomes: no pregnancy, miscarriage and live birth. The decision tree model analysis provided us with options for no pregnancy, miscarriage, or live birth in each scenario. The CLBR was the major outcome/validity of this model. See “Supplementary Information” and “Supplementary Table S1” for additional details regarding the calculation of probabilities and costs.

### Sensitivity analysis

One-way sensitivity analysis is performed to ensure the robustness of the cost-effectiveness analysis results by adjusting one parameter to the highest or lowest value at a time. Assume that each input parameter varies independently. The cost expenditure is set to fluctuate within a range of − 20 to + 20%. The live birth rate of fresh embryo transfer for the GnRHa group was estimated to achieve comparable cost-effectiveness between the two protocols. A tornado chart was used to show the results of the influence variables and one-way sensitivity. The parameters used in the sensitivity analysis are listed in Supplementary Table S1 in the supplementary information.

### Statistical analyses

The Shapiro test was used to determine the normality of all continuous variables. Variables with a normal distribution are denoted by $\overline{x }\pm s$. Student's t test was used for statistical comparisons. Variables that did not have a normal distribution are presented as the median ± interquartile range (IQR), and the Mann‒Whitney U test was used to analyze differences between groups. The categorical variables are described in terms of n (%), the difference test was the chi-square test, and Fisher’s exact probability test was used when the predicted number was small.

Because of the large difference in sample sizes between the two groups, patients with similar features were selected through propensity score matching (PSM) with nearest neighbor matching and a caliper of 0.02. Female age (years), infertility duration (years), body mass index (BMI, kg/m2), anti-Müllerian hormone (AMH) level, basal follicle stimulation hormone (bFSH, IU/L) level, and infertility cause were the factors utilized for matching. Subclassification and nearest neighbor matching are the methods for selecting a matching object. Comparisons of important baseline characteristics between groups were not statistically significant. A logistic regression model was used to analyze the influencing factors that affect the live birth rate, a Kaplan‒Meier curve was used to develop the cumulative live birth curve for the two protocols, and the log-rank test was utilized for the difference test. The factors influencing the cumulative live birth rate were analyzed with a Cox proportional hazards model.

Statistical analyses were performed using the Statistical Program for Social Sciences (SPSS Inc., Version 24.0, Chicago) and R version 4.1.0 (R Core Team, Vienna, Austria). A 2-tailed value of *P* < 0.05 was considered to indicate statistical significance.

### Ethical approval

The study was conducted in accordance with the Declaration of Helsinki. The study was approved by the Medical Ethical Committee of Union Hospital (No. 2023-S0455) and registered in the Chinese Clinical Trial Registry (ChiCTR2300076537).

### Informed consent

The need for informed consent was waived by the Medical Ethical Committee of Union Hospital because this was a retrospective study.

## Results

Between January 2016 and June 2021, 11,809 IVF/ICSI cycles were performed in our center. There were 2871 cycles that met the inclusion criteria but did not meet the exclusion criteria, with 1074 cycles using the GnRHa protocol and 1797 cycles using the CC cotreatment protocol. The detailed flow chart of this study is shown in Supplementary Fig. S2 in the supplementary information.

### Comparisons of baseline characteristics between the two groups

Before matching, there were significant differences in female age, infertility duration, infertility type, basal AMH level, basal serum FSH level, infertility cause, and insemination method between the CC cotreatment and GnRHa groups. To reduce bias caused by differences in baseline characteristics between the two groups, PSM was performed at a 1:1 ratio using the 9 aforementioned variables. After matching, 375 cycles were assigned to the GnRHa and CC cotreatment groups, with no significant difference in baseline characteristics between the matched groups. A comparison of the baseline characteristics between the two groups before and after matching is shown in Table 1.

**Table 1:** Baseline characteristics of the women in the CC cotreatment and GnRHa groups before and after matching.

Characteristic	Before propensity score matching			After propensity score matching		
	CC cotreatment group (n = 1,797)	GnRHa group (n = 1,074)	*p* value	CC cotreatment group (n = 375)	GnRHa group (n = 375)	*p* value
Maternal age (y)	39 (37, 42)	36 (35, 38)	< 0.001	37 (36, 39)	37 (36, 39)	0.410
Infertility duration (y)	4 (2, 8)	3 (2, 7)	0.023	3 (2, 7)	4 (2, 8)	0.268
Primary infertility, n (%)	381 (21.20)	349 (32, 50)	< 0.001	99 (26.40)	110 (29.33)	0.415
Cycle number, n (%)			< 0.001			1
First cycle	1116 (62.10)	909 (84.64)		279 (74.40)	278 (74.10)	
Repeated cycles	681 (37.90)	165 (15.36)		96 (25.60)	97 (25.90)	
Basal FSH level (IU/L)	8.36 (6.77, 10.92)	6.70 (5.68, 7.96)	< 0.001	7.12 (6.01, 9.07)	7.24 (6.13, 8.21)	0.376
Basal LH level (IU/L)	4.51 (3.36, 6.15)	4.75 (3.50, 6.56)	0.001	5.09 (3.47, 6.42)	5.15 (3.65, 6.45)	0.340
AMH (ng/ml)	1.09 (0.63, 1.74)	3.43 (2.22, 5.23)	< 0.001	1.45 (0.94, 2.13)	1.35 (1.01, 2.55)	0.270
Body mass index (kg/m^2^)	22.60 (20.80, 24.70)	22.71 (20.80, 25.03)	0.189	22.91 (21.13, 25.12)	22.88 (21.01, 24.93)	0.237
Cause of infertility, n(%)			< 0.001			0.956
Tubal	810 (45.08)	376 (35.01)		145 (38.67)	133 (35.47)	
Male factor	251 (13.97)	106 (9.87)		29 (7.73)	35 (9.33)	
Anovulatory	57 (3.17)	39 (3.63)		35 (9.33)	42 (11.20)	
Unexplained	294 (16.36)	327 (30.45)		98 (26.13)	104 (27.73)	
Diminished ovarian reserve	304 (16.92)	181 (16.85)		40 (10.67)	39 (10.40)	
Endometriosis	66 (3.67)	40 (3.72)		22 (5.87)	16 (4.27	
Mixed factors	15 (0.83)	5 (0.47)		6 (1.60)	6 (1.60)	
Insemination method, n(%)			< 0.001			0.230
IVF	916 (50.97)	714 (66.48)		229 (61.07)	250 (66.66)	
ICSI	818 (45.52)	320 (29.80)		133 (35.47)	111 (29.60)	
IVF + RICSI	63 (3.51)	40 (3.72)		13 (3.47)	14 (3.73)	

Data are presented as the median (25th and 75th percentile) or number (%). Mann‒Whitney U statistics were used for continuous variables and chi-square tests were used for categorical variables. There were no significant differences after propensity score matching.

*CC cotreatment* clomiphene citrate and gonadotropin cotreatment, *GnRHa* gonadotropin-releasing hormone agonists, *FSH* follicle-stimulating hormone, *LH* luteinizing hormone, *AMH* anti-müllerian hormone, *IVF* in vitro fertilization, *ICSI* intracytoplasmic sperm injection, *RICSI* early rescue intracytoplasmic sperm injection.

### Comparisons of ovarian stimulation and embryo laboratory outcomes

The rate of cycle cancellation in the CC cotreatment group was significantly greater than that in the GnRHa group (13.07% vs. 8.00%, *p* = 0.032). However, this was mainly due to the implementation of blastocyst embryo culture and the freeze-all strategy in the CC cotreatment group, which resulted in more patients in this group having no available embryos after in vitro blastocyst culture (6.52% vs. 2.55%, *p* = 0.011). Moreover, the number of cycles of ovarian stimulation that failed due to ovarian nonresponse or poor response, premature ovulation and oocyte retrieval failure was not significantly different (all *p* > 0.05). There was no significant difference in the duration of ovarian stimulation or the total gonadotropin dosage between the two groups. Premature LH surge was more common in the CC cotreatment group than in the GnRHa group (6.13% vs. 0.53%, *p* < 0.001), although there was no difference in the premature ovulation rate between the two groups (Table 2).

**Table 2:** Ovarian stimulation and embryo laboratory outcomes of the two groups after propensity score matching.

Characteristic	CC cotreatment group	GnRHa group	*p* value	Risk ratio (95% CI)
Patients, n	375	375	–	–
Failure of pituitary downregulation, %(n)	–	1.33 (3/375)	–	–
OS failure due to ovarian nonresponse or poor response, %(n)	2.13 (8/375)	1.60 (6/375)	0.589	1.34 (0.46–3.90)
Premature LH surge, %(n)	6.13 (23/375)	0.53 (2/375)	< 0.001	12.19 (2.85–52.07)
Premature ovulation, %(n)	2.40 (9/375)	0.80 (3/375)	0.081	3.05 (0.82–11.35)
No oocyte retrieved cycles during OPU, %(n)	2.40 (9/375)	2.13 (8/375)	0.806	1.13 (0.43–2.96)
No. of cycles without available embryo after in vitro culture, %(n)	6.52 (23/375)	2.40 (9/375)	0.011	2.66 (1.21–5.82)
Total no. of cycle cancellations, %(n)	13.07 (49/375)	8.00 (30/375)	0.032	0.58 (0.36–0.93)
Did not undergo embryo transfer, %(n)	1.53 (5/326)	2.61 (9/345)	0.330	0.58 (0.19–1.75)
LH on trigger day (mIU/mL)	7.83 (4.92, 10.49)	2.81 (2.29, 3.48)	< 0.001	–
E2 on trigger day (pg/ml)	1447 (884.2, 2268)	1609 (936, 2516)	0.18	–
Progesterone on trigger day (ng/ml)	0.97 (0.64, 1.38)	0.95 (0.70, 1.24)	0.728	–
Gn dosage (IU)	2495 (2025, 3075)	2400 (2000, 3088)	0.356	–
Gn duration, d	10 (9, 12)	10 (9, 12)	0.071	–
Moderate or severe OHSS, %(n)	1.86 (7/375)	2.40 (9/375)	0.801	1.29 (0.48–3.51)
Average no. of oocytes retrieved	6 (4, 10)	11 (7, 11)	< 0.001	–
Average no. of ≥ 14 mm follicles	6 (4, 9)	9 (7, 12.5)	< 0.001	–
Mature oocyte rate, %	88.9 (75.00, 100)	85.7 (72.73, 100)	0.001	–
2PN fertilization rate, %	75.00 (60.00, 100)	75.00 (57.33, 87.40)	0.255	–
Cleavage rate, %	100 (100, 100)	100 (100, 100)	0.147	–
High-quality D3 embryo rate, %	37.50 (0, 60.00)	33.33 (1.11, 50.00)	0.092	–
Total no. of available embryos on Day 3	4 (2,6)	5 (3,7)	0.045	–
Available embryo rate, %	80.00 (60.00, 90.00)	80 (60.00, 90.00)	0.211	–

Data are presented as the median (25th and 75th percentile) or number (%). Mann‒Whitney U tests were used for continuous variables, and chi-square tests were used for categorical variables.

*CC cotreatment* clomiphene citrate and gonadotropin cotreatment, *GnRHa* gonadotropin-releasing hormone agonist, *CI* confidence interval, *OS* ovarian stimulation, *OPU* oocyte pick-up, *LH* luteinizing hormone, *E2* estradiol, *P* progesterone, *Gn* gonadotropin, *OHSS* ovarian hyperstimulation syndrome, *2PN* two pronuclei.

A premature LH surge was defined as a serum LH level ≥ 10 mIU/mL or an increase of more than 2.5 times above the basal level during ovarian stimulation.

The total number of cycle cancellations was calculated as follows: pituitary downregulation (in the GnRHa group) + OS failure cycles + cycles with no oocytes retrieved + cycles without available embryos after in vitro culture + cycles cancelled for personal or other reasons.

The percentage of mature oocytes was calculated as the total number of mature oocytes divided by the total number of retrieved oocytes × 100%

2PN fertilization rate = the number of 2PN oocytes on Day 1/(total number of IVF fertilized oocytes + total number of MII oocytes injected) × 100%

D3 high-quality embryo rate = the high-quality embryo number on Day 3/normal fertilization cleavage embryo number × 100%

The available embryo rate = the number of embryos available for frozen embryo transfer or blastocyst culture/the number of normally fertilized oocytes.

According to the outcomes from the embryo laboratory, the CC cotreatment group exhibited significantly fewer ≥ 14 mm follicles and retrieved oocytes [6 (4, 9) vs. 9 (7, 12.5); 6 (4, 10) vs. 11 (7, 11), respectively; both p < 0.001], along with a notably higher rate of oocyte maturation [88.89% (75%, 100%) vs. 85.71% (72.73%, 100%), *p* = 0.001] compared to the GnRHa group. However, there were no significant differences in the normal fertilization rate, cleavage rate or top-quality embryo rate between the two groups. Due to the lower number of retrieved oocytes in the CC cotreatment group than in the GnRHa group, the former had significantly fewer available embryos [4 (2, 6) vs. 5 (3, 7), *p* = 0.045]. However, there was no difference observed in the percentage of available embryos between the two groups (Table 2).

## Comparisons of clinical outcomes between the two groups

During the two-year study period, 445 FET cycles were performed in the CC cotreatment group. A total of 562 transfer cycles were conducted in the GnRHa group (214 fresh embryo transfers and 348 FETs). Because blastocyst culture and the freeze-all strategy were implemented in the CC cotreatment group, the embryo implantation rate (45.42% vs. 39.46%, *p* = 0.028) and the clinical pregnancy rate per transfer (52.36% vs. 46.02%, *p* = 0.049) were significantly greater in the CC cotreatment group than in the GnRHa group. However, there were no significant differences in the rates of multiple pregnancy, miscarriage or ectopic pregnancy per transfer between the two groups (all *p* > 0.05). The incidence of moderate or severe OHSS was not significantly different between the two groups (Table 3).

**Table 3:** Pregnancy and cumulative outcomes of the CC cotreatment group and GnRHa group after propensity score matching.

Characteristic	CC cotreatment group	GnRHa group	*p* value	Risk ratio (95% CI)
Patients, n	375	375	–	
Total no. of patients who underwent embryo transfer	321	336	–	
Total no. of embryos transferred	579	821	–	–
Total no. of transfer cycles	445	565	–	–
Cycle type of embryo transfer, n (%)				
Fresh embryo transfer	–	217	–	
Frozen embryo transfer	445	348	–	
Stage of embryo transferred, n (%)				
Day 3 embryo transfer	23 (3.97)	376 (45.80)	< 0.001	0.05 (0.03–0.08)
Blastocyst transfer	556 (96.03)	445 (54.20)		
No. of embryos transferred, n (%)				
1	311 (69.89)	309 (54.69)	< 0.001	1.92 (1.48–2.50)
2	134 (30.11)	256 (45.31)		
Implantation rate, %(n)	45.42 (263/579)	39.46 (324/821)	0.028	1.27 (10.3–1.58)
Clinical pregnancy rate per transfer cycle, %(n)	52.36 (233/445)	46.02 (260/565)	0.045	1.29 (1.01–1.65)
Multiple birth rate, %(n)	17.17 (40/233)	16.92 (44/260)	0.943	1.02 (0.64–1.63)
Miscarriage rate, %(n)	29.61 (69/233)	33.85 (88/260)	0.314	0.82 (0.56–1.20)
Ectopic pregnancy rate, %(n)	0.43 (1/233)	0.77 (2/260)	1.000	0.56 (0.05–6.17)
Cumulative biochemical pregnancy rate per initial cycle, %(n)	60.00 (225/375)	68.80 (258/375)	0.015	0.68 (0.50–0.92)
Cumulative clinical pregnancy rate per initial cycle, %(n)	51.73 (194/375)	60.27 (226/375)	0.019	0.71 (0.53–0.94)
CLBR per initial cycle, %(n)	41.07 (154/375)	45.33 (170/375)	0.238	0.84 (0.63–1.12)
Preterm birth rate, %(n)	8.44 (13/154)	14.71 (25/170)	0.080	0.54 (0.26–1.09)
Average birth weight	3300 (2973, 3550)	3280 (2650, 3600)	0.143	–
Average gestational age at birth	38 (38, 39)	38 (37, 39)	0.005	–
Congenital malformation rates	0	0	1.000	–
TTLB	360 (315.8, 445)	326.5 (257.5, 436)	< 0.001	–

Data are presented as the median (25th and 75th percentile) or number (%). Mann‒Whitney U tests were used for continuous variables, and chi-square tests were used for categorical variables.

*CC cotreatment* clomiphene citrate and gonadotropin cotreatment, *GnRHa* gonadotropin-releasing hormone agonist, *CI* confidence interval, *CLBR* cumulative live birth rate, *TTLB* time to first live birth.

**Figure 2:**
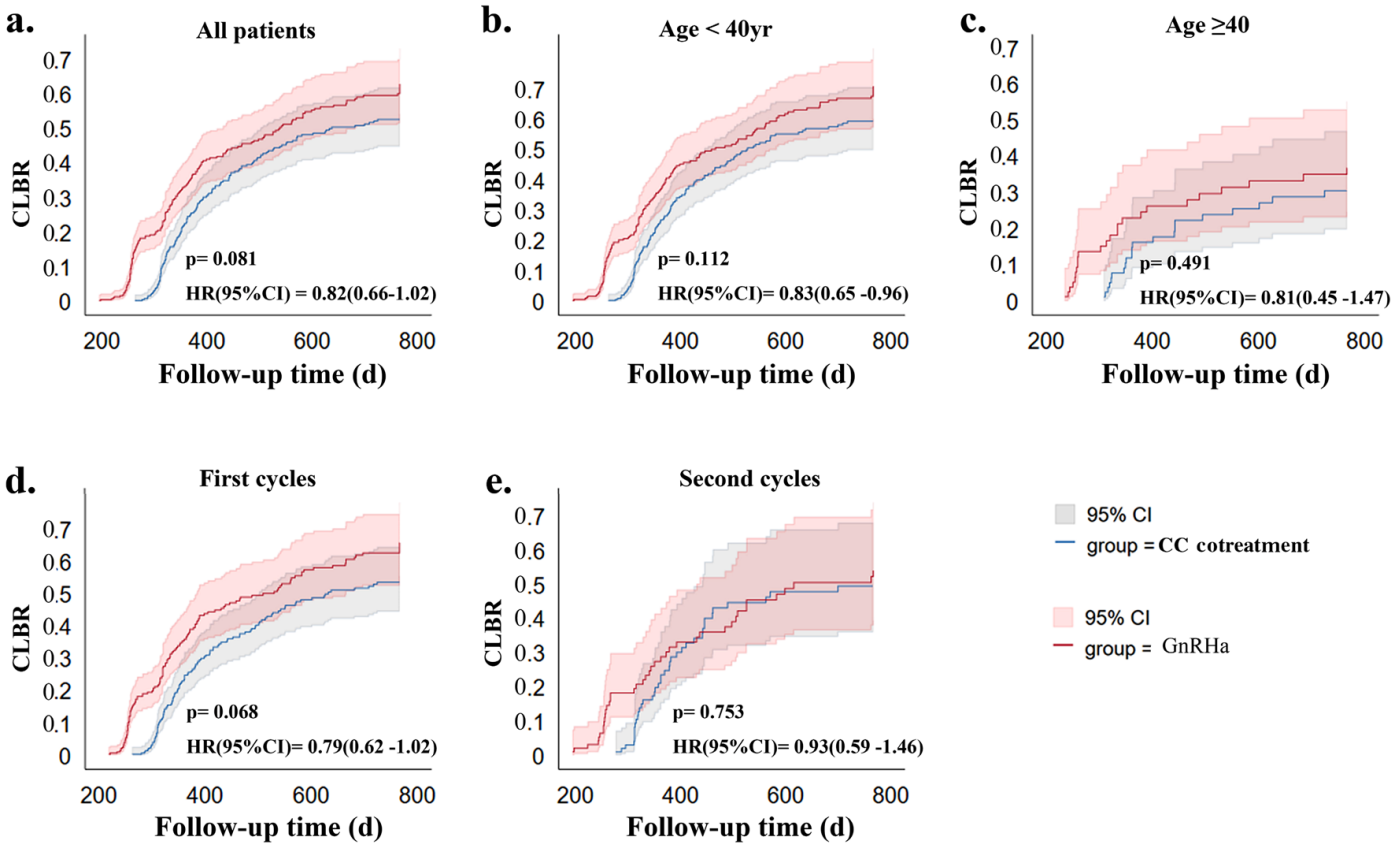
Kaplan–Meier curves of the cumulative live birth rate (CLBR) in patients receiving the CC cotreatment or GnRHa protocol. (**a**) CLBR of all the patients; (**b**) CLBR in women aged > 35 and < 40 years; (**c**) CLBR in women aged ≥ 40 years; (**d**) CLBR in women undergoing their first IVF cycle; (**e**) CLBR in women undergoing their second IVF cycle. CI, confidence interval; CC cotreatment, clomiphene citrate and gonadotropin cotreatment; GnRHa, gonadotropin-releasing hormone agonist; HR, hazard ratio.

To determine the cumulative clinical outcomes over 24 months of follow-up, the total number of initial cycles was used as the denominator. The CC cotreatment group had a significantly lower cumulative clinical pregnancy rate than did the GnRHa group (51.73% vs. 60.27%, *p* = 0.023). The CLBR, however, did not differ significantly between the two groups (41.07% vs. 45.33%, *p* = 0.269) (Table 3). Furthermore, no significant difference in the cumulative live birth rate was observed between the two groups in the Kaplan‒Meier analysis at the final follow-up point (24 months) (*p* = 0.081, HR (95% CI) = 0.82 (0.66‒1.02)) (Fig. 2). There were no significant differences in gestational age, birth weight, or the preterm birth rate between the two groups. The time to live birth (TTLB) was calculated from the day of oocyte retrieval to the day of live birth. The TTLB in the CC cotreatment group was considerably longer than that in the GnRHa group [360 (315.8, 445) vs. 326.5 (257.5, 435.8), p = 0.001], and the median time to live birth in the CC cotreatment group was approximately 33.5 days later (Table 3).

A binary logistic regression analysis was performed, which included the following: female age, infertility type, duration of infertility, number of IVF cycles, BMI, basal FSH level, AMH level, total gonadotropin dosage, duration of ovarian stimulation, number of oocytes retrieved, number of available embryos, infertility cause, and ovarian stimulation protocol. After controlling for other confounding factors, female age, AMH level, and the number of available embryos were found to be significantly associated with the CLBR. However, the ovarian stimulation protocol (the CC cotreatment protocol or the GnRHa protocol) had no effect on the CLBR (hazard ratio (HR) 95% CI 1.475 (0.994–2.187); *p* = 0.084) (Table 4).

**Table 4:** Regression analysis of factors for the prediction of a cumulative live birth for the fresh plus all frozen embryo transfer cycles.

Factors predicting a cumulative live birth	Coefficient(B)	S.E	Wald (χ^2^)	P value	Hazard ratio (95% CI)
Maternal age	− 0.135	0.039	11.877	0.001	0.874 (0.809–0.943)
Type of infertility					
Primary infertility					1
Secondary infertility	− 0.052	0.198	0.069	0.794	0.949 (0.644–1.400)
Infertility duration	0.000	0.022	0.000	0.988	1.000 (0.958–1.043)
Cycle number					
The first cycle					1
The second cycle	0.084	0.200	0.175	0.676	1.087 (0.734–1.610)
Treatment protocol					
GnRHa					1
CC cotreatment	0.388	0.201	3.725	0.084	1.475 (0.994–2.187)
Body mass index	0.000	0.000	0.160	0.689	1.000 (1.00–1.00)
Basal FSH	0.026	0.035	0.545	0.461	1.026 (0.958–1.098)
AMH	0.116	0.041	18.77	< 0.001	1.112 (1.017–1.431)
Total Gn dosage	0.000	0.000	0.570	0.450	1.000 (1.00–1.00)
Days of stimulation	− 0.106	0.073	2.102	0.147	0.900 (0.780–1.038
No. of oocytes retrieved	0.023	0.020	1.378	0.240	1.023 (0.985–1.063)
No. of available embryos	0.352	0.050	50.517	< 0.001	1.422 (1.290–1.567)
Cause of infertility					
Tubal					1
Male factor	0.439	0.307	2.043	0.153	1.551 (0.850–2.830)
Anovulatory	− 0.460	0.456	1.017	0.313	0.631 (0.258–1.543)
Unexplained	0.087	0.210	0.173	0.678	1.091 (0.723–1.657)
Diminished ovarian reserve	0.055	0.308	0.032	0.857	1.057 (0.578–1.933)
Endometriosis	0.371	0.420	0.782	0.376	1.449 (0.637–3.298)
Mixed	0.689	0.741	0.865	0.352	1.991 (0.466–8.506)

*CC cotreatment* clomiphene citrate and gonadotropin cotreatment, *GnRHa* gonadotropin-releasing hormone agonist, *FSH* follicle-stimulating hormone, *AMH* anti-müllerian hormone, *Gn* gonadotropin, *CI* confidence interval.

### Subgroup analysis

Subgroup analysis was conducted based on age (≥ 40 or < 40 years) and cycle number (1st or 2nd cycle). The Kaplan‒Meier subgroup analysis results also demonstrated that at the last follow-up time point (24 months), there was no statistically significant difference in the CLBR between the CC cotreatment protocol and the GnRHa protocol, regardless of age or cycle number (all *p* values > 0.05). (Fig. 2a–e). Particularly in the 2nd cycle subgroup, the differences in the CLBR between the CC cotreatment group and the GnRHa group were even more marginal (Fig. 2e).

### Cost-effectiveness analysis

The average cost per patient in the CC cotreatment group was 21.27% less than that in the GnRHa group (¥32,301.42 vs. ¥39,174.22). The cost per live birth in the CC cotreatment group was ￥79,367.07, while the cost per live birth in the GnRHa group was ￥84,996.05. Compared with that in the CC cotreatment group, the cumulative live birth rate in the GnRHa group was greater, but the incremental cost-effectiveness ratio (ICER) for each additional live birth was ￥127,493.9.

**Figure 3:**
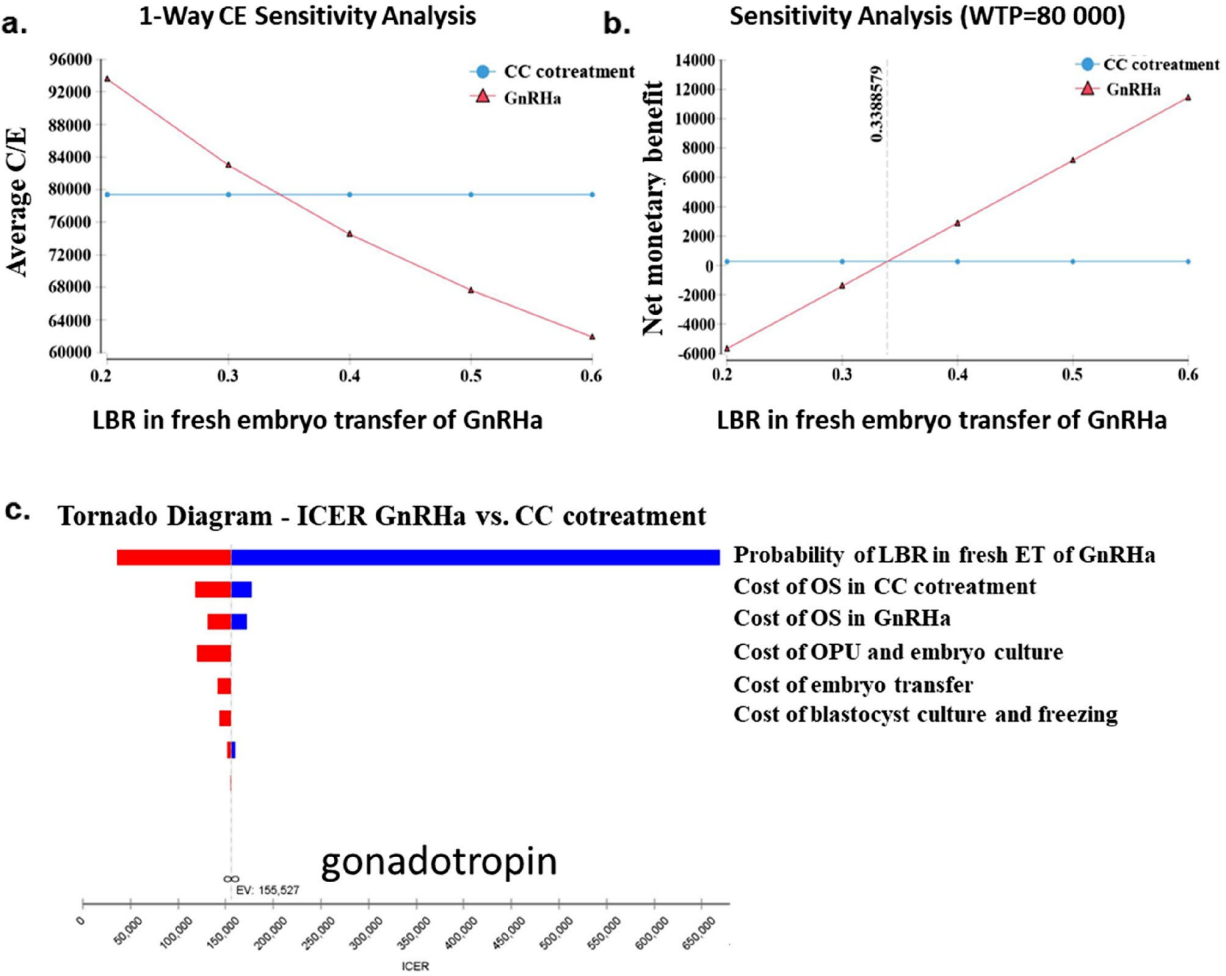
One-way sensitivity analysis of the cost-effectiveness ratio between the CC cotreatment and GnRHa protocols. (**a**) One-way C/E sensitivity analysis. (2) One-way NMB sensitivity analysis. (3) Tornado diagram. The parameters affecting the ICER are shown. CC cotreatment, clomiphene citrate and gonadotropin cotreatment ovarian stimulation; GnRHa, gonadotropin-releasing hormone agonist; LBR, live birth rate; OS, ovarian stimulation; OPU, oocyte pick-up; ICER, incremental cost-effectiveness ratio; NMB, net monetary benefit.

When the live birth rate of fresh embryo transfer in the GnRHa group increased to 33.89%, the cost per live birth in the GnRHa group was comparable to that in the CC cotreatment group according to one-way sensitivity analysis. The tornado chart of the one-way sensitivity analysis revealed that the live birth rate after fresh embryo transfer and the cost of ovarian stimulation were the most important factors influencing the ICER. All of these data indicate that our model is very robust (Fig. 3).

## Discussion

After ovarian stimulation, retrieving a specific number and quality of oocytes is critical for IVF success. The largest barrier to achieving an optimal IVF live birth rate in women over the age of 35 years is the aging-related reduction in oocyte development potential and/or oocyte number. Over the last two decades, older patients have been subjected to various ovarian stimulation protocols, as well as complementary drugs^29,30^ such as growth hormone, recombinant luteinizing hormone, dehydroepiandrosterone, and coenzyme Q10. However, the efficacy and cost-effectiveness of each treatment have yet to be determined. In this retrospective PSM cohort study of infertile women over the age of 35 years, we found no significant difference in the CLBR between the CC cotreatment group and the conventional GnRHa group (41.07% vs. 45.33%). The TTLB for women who received the CC cotreatment protocol increased, but the cost per live birth was significantly lower than that for those who received the conventional GnRHa protocol.

In the present study, we first compared oocyte and embryo quality between the CC cotreatment and GnRHa groups after PSM and discovered that while the number of ≥ 14 mm follicles and retrieved oocytes was lower in the CC cotreatment group, the oocyte maturity rate was significantly higher. The significantly lower number of retrieved oocytes in the CC cotreatment group than in the GnRHa group could be due to pituitary suppression in the GnRHa group, which resulted in more uniform follicle sizes at the initial stage and improved synchronization during follicular development. In the present study, the CC cotreatment group had a lower oocyte yield than the GnRHa group, which is consistent with previous studies comparing GnRHa protocols and non-GnRHa protocols^31,32^. Notably, there were no significant differences between the two groups in terms of the normal fertilized oocyte rate, high-quality embryo rate or available D3 embryo rate, suggesting that there was no difference in oocyte competence or embryonic development potential. This finding is consistent with the findings of Bhor SA et al., who revealed that ovarian stimulation with CC and gonadotropin had no effect on the blastocyst formation rate compared to a GnRH antagonist strategy^20^. Furthermore, the use of CC to induce ovulation has been reported in PCOS animal models to reverse insulin-like growth factor family gene and transforming growth factor family gene expression disorders in the ovum, increase oocyte maturation, and improve in vitro maturation IVF treatment outcomes^33^.

In the present study, due to the continuous use of CC in the CC cotreatment group until the trigger day and considering the influence of CC on endometrial receptivity, the CC cotreatment group underwent blastocyst culture and a “freeze-all” strategy. This also explains why the TTLB in the CC cotreatment group was much longer than that in the GnRHa group. Moreover, it was not possible to directly compare the pregnancy outcomes of single embryo transfer under the two protocols; hence, the CLBR of one IVF cycle was chosen as one of the primary outcome measures in this study. Our results showed that the CC cotreatment group had greater rates of embryo implantation and clinical pregnancy per transfer; therefore, even though this group had a greater rate of cycle cancellation, there was no significant difference in the CLBR per initial cycle between the two groups. In addition, there were no significant differences in gestational age, average weight, the preterm birth rate or the incidence of congenital malformations between the two groups. The results showed that the CC cotreatment protocol did not have adverse effects on embryonic development potential or progeny health. A recent retrospective study also showed that recent (< 90 days) CC exposure did not affect the implantation potential of transferred mono-euploid embryos^34^. Furthermore, a retrospective cohort analysis with a small sample size revealed that, compared to the use of an antagonist protocol, the use of a CC or gonadotropin protocol had no influence on the live birth rate after frozen embryo transfer^20^. In terms of the cumulative live birth rate and progeny safety, the CC cotreatment protocol is equivalent to the GnRHa protocol in infertile women over the age of 35 years.

The rate of ART utilization is largely related to the affordability and accessibility of the technology^35^. The high cost of IVF is a major reason why many couples discontinue treatment^36–38^. Multiple cycles of ovarian stimulation and oocyte retrieval may be required in older women due to a decrease in the quantity of oocytes retrieved or in oocyte competence, which increases the cost. As a result, when selecting the right ovarian stimulation protocol for these patients, not only the success rate but also the cost-effectiveness of the protocol should be taken into account^38^. The cost of various ovarian stimulation protocols might vary greatly depending on the drug dosage and the number of embryo transfer cycles. For example, mild-stimulation protocols, which have been studied more frequently in older IVF patients in recent years, may have a higher average cost per live birth than conventional protocols due to their low oocyte retrieval rate per cycle and high cycle cancellation rate, which often necessitate multiple cycles of ovarian stimulation and embryo transfer^39^. As a result, some physicians believe that retrieving a sufficient number of mature oocytes in a single ovarian stimulation cycle is necessary. Blastocyst culture, single blastocyst transfer and vitrification of excess embryos after oocyte retrieval are less expensive and more efficient than “unconventional” protocols such as the natural cycle, modified natural cycle, aromatase inhibitor/low-dose FSH protocol, and CC/low-dose FSH protocol^40^. In the present study, we found that the cost per live birth in the CC cotreatment group was significantly lower than that in the GnRHa group. The observed outcome can be attributed to two significant factors. First, the CC cotreatment group received CC in combination with gonadotropin (Gn) for ovarian stimulation, which significantly reduced pharmaceutical expenditures. Second, patients in the CC cotreatment group underwent blastocyst culture and a freeze-all strategy, resulting in fewer embryo transfer cycles per live birth. Satwik et al.^41^ also showed that using a combination of CC and Gn for ovarian stimulation can lower the average cost of IVF therapy. According to their study, patients with an AMH level < 1.4 ng/ml experienced a 13–28% cost reduction when CC and Gn were used together for ovarian stimulation. The CC cotreatment protocol is less expensive than the GnRHa protocol for women over the age of 35 years, which justifies its use in this age group.

In this study, we carried out further subgroup analysis based on female age and number of cycles, and the results showed no significant difference in the CLBR between subgroups regardless of whether the CC cotreatment or GnRHa protocol was used. Especially in the 2nd cycle subgroup comparison, the CLBR curves of the two protocols almost overlapped. Since many patients who undergo repeated IVF cycles have an unexpectedly poor ovarian response or poor embryo quality, several studies have reported that the addition of CC therapy in repeat IVF cycles can improve the ovarian response and minimize the cycle cancellation rate^42,43^. Based on these results, we believe that the application of the CC cotreatment protocol in older patients undergoing repeated IVF cycles deserves further investigation in future prospective studies.

To our knowledge, this is the first study to compare a CC-based ovarian stimulation protocol with the GnRHa protocol to investigate the CLBR, TTLB, and cost-effectiveness of the CC-based protocol. Doctors should consider cost-effectiveness when recommending ovarian stimulation to patients. Second, we included nonselected infertile couples older than 35 years and calculated the CLBR of one IVF cycle 24 months after oocyte retrieval; none of the patients were lost to follow-up. Our cohort's results are therefore applicable to the general population above the age of 35 years. The other advantage of the present study was the use of PSM to balance the differences in baseline characteristics between the CC cotreatment and GnRHa groups. This is because selection bias and the imbalance of baseline characteristics between groups are very common problems in observational studies^44^. The main advantage of propensity score matching (PSM) is its capacity to eliminate potential bias between treatment and control groups in observational studies, improving internal validity and enabling more reliable outcome comparisons^45^.

There are some limitations to the present study. First, this was a single-center retrospective study, and a variety of potential confounding factors could have affected the results. Although the PSM method was adopted in this study to match 9 baseline characteristics at a 1:1 ratio and eliminate the influence of confounding factors as much as possible, some known or unknown confounding factors may still affect the statistical results. Second, during the study period, the main ovarian stimulation protocols for older patients in our center were the CC cotreatment and GnRHa protocols, and a few patients received GnRH antagonists and a mild stimulation protocol. Hence, in the present study, we were unable to directly compare the CC cotreatment protocol with other ovarian stimulation protocols. Third, because some patients did not undergo a baseline ultrasound examination at our clinic at initiation, we lack data on antral follicle count (AFC) for both groups, potentially affecting their comparability. However, in PSM, we included ovarian reserve variables such as AMH, bFSH, and age to ensure balanced ovarian reserve status. Therefore, despite the missing AFC data, we believe it's unlikely to significantly impact our study's conclusions.

## Conclusions

This study revealed that the cumulative live birth rates among older women receiving the CC cotreatment protocol were similar to those receiving the GnRHa protocol. Despite the CC cotreatment group having a longer TTLB than the GnRHa group, the economic cost per live birth was significantly lower in the CC cotreatment group. This suggests that CC cotreatment is a viable option for ovarian stimulation in older women undergoing IVF.

### Supplementary Information

Supplementary Information.

## Data Availability

The data presented in this study are available upon request from the corresponding author.
