# Supplementary material for: The cumulative live birth rate and cost-effectiveness of the clomiphene and gonadotropin cotreatment protocol versus the mid-luteal GnRH agonist protocol in women over 35 years old
Source: Sci Rep. 2024 Jun 5;14:12894. doi: 10.1038/s41598-024-63842-x (PMC11153521; doi:10.1038/s41598-024-63842-x)
Supplement: Supplementary file 1 — Supplementary Information. [file 41598_2024_63842_MOESM1_ESM.pdf]

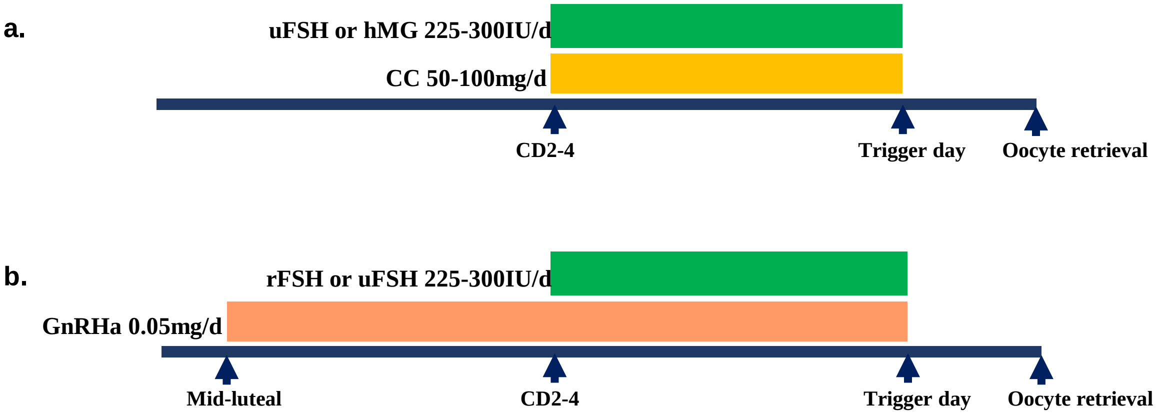

Supplementary figure S1. Schematic diagram of the two ovarian stimulation protocols. **a.** Schematic diagram of ovarian stimulation in CC cotreatment protocol: from the second or third day of menstruation until the trigger day, 100mg of CC (Fertila, Codal Synto Ltd., Cyprus) was administered orally; 225-300IU of uFSH were also given daily, with the dosage being adjusted based on ovarian response. When bilateral ovaries had ≥2~3 follicles with diameter >18mm, 10, 000 IU human chorionic gonadotropin (hCG) (Lizhu Pharmaceutical Trading Co., China) or 250mg Ovidrel (Merck Serono S.p.A., Modugno, Italy) was administered to trigger the final maturation of the oocytes. Oocytes were retrieved 34~36h later. b**.** Schematic diagram of ovarian stimulation in GnRHa protocol: Tripreilin acetate was injected 0.05mg/d (Ferring Pharmaceuticals, SaintPrex, Switzerland) at the mid-luteal phase of the previous cycle for pituitary down-regulation. When the down-regulation standard was reached (serum luteinizing hormone (LH) < 5IU/L, serum estradiol (E2) < 50pg/ml, endometrial thickness <10mm, no functional ovarian cyst, urine follicle-stimulating hormone (uFSH) (Zhuhai Lizon Pharmaceutical, Zhuhai, China) or recombinant follicle-stimulating hormone (rFSH) (Merck Serono, Buchs, Switzerland) was initiated with 225-300U, and the adjustment was made according to the ovarian response. CD: cycle day. CC: clomiphene citrate. uFSH: urine follicle-stimulating hormone. rFSH: recombinant follicle-stimulating hormone; GnRHa, gonadotropin-releasing hormone agonist.

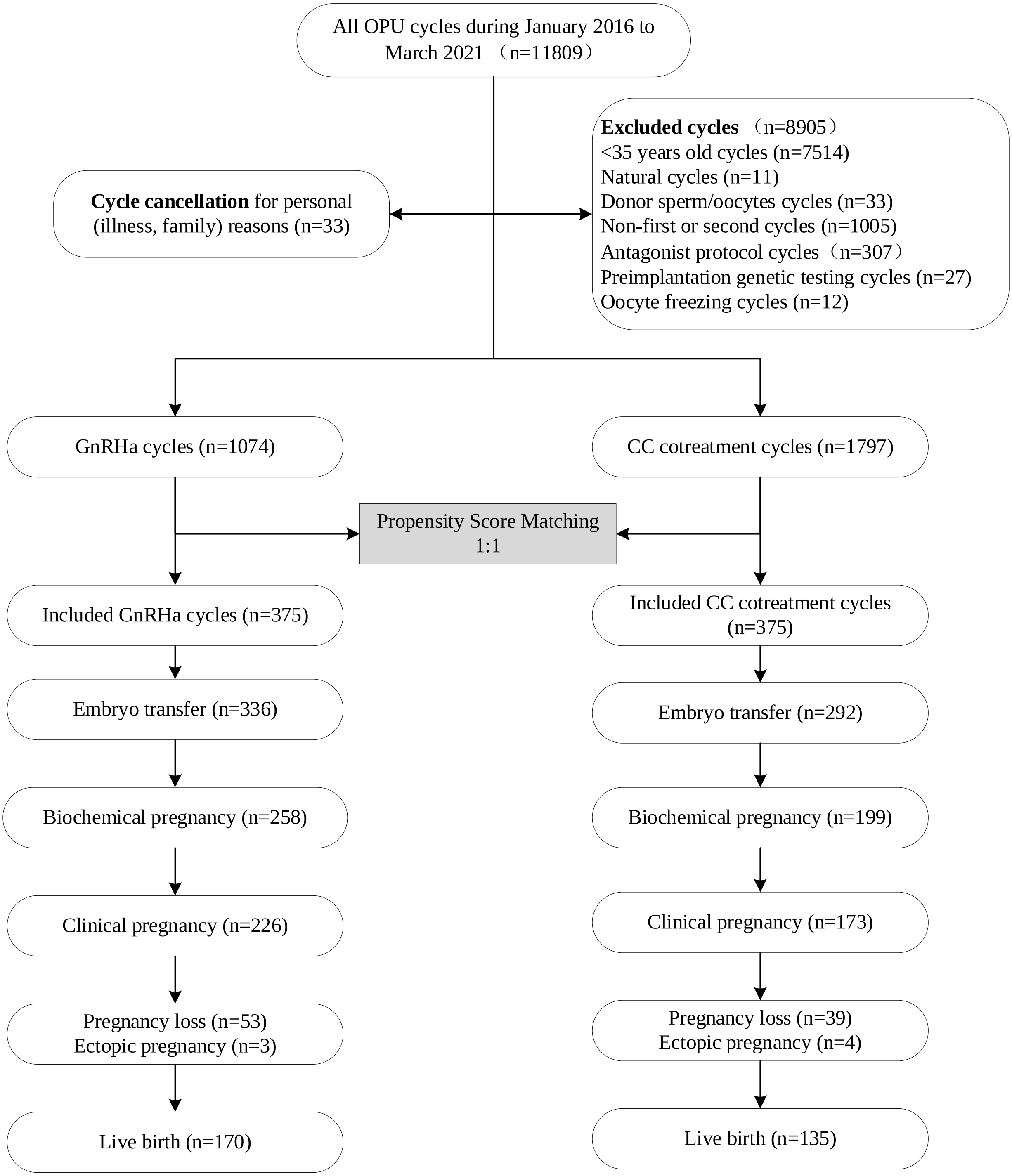

Supplementary figure S2. The study flow chart. OPU, oocyte pick-up; CC cotreatment, clomiphene citrate and gonadotropin cotreatment; GnRHa, Gonadotropin-releasing hormone agonists.

Supplementary Table S1. Decision tree model input parameters.

Parameter	Distribution	Value	One way sensitivity analysis min	max
**Cost, CNY**				
pre-IVF examination	Gamma	5000	3750	5250
OS with CC cotreatment protocol [a]	Gamma	5523.86	4812.11	6586.735
OS with GnRHa protocol [a]	Gamma	12333.89	10710.68222	15105.48631
Oocyte retrieval and embryo culture	Gamma	9175	7675	11468.75
Fresh embryo transfer and lueteal [b] support	Gamma	3524.38	2643.285	4405.475
Frozen embryo transfer and lueteal support [b]	Gamma	5677.38	4258.035	7096.725
Misscarriage [c]	Gamma	4090.76	3068.07	5113.45
Live birth [c]	Gamma	11272.28	9272.28	16272.28
**Probability**				
Live birth rate in 1st FET cycle of GnRHa group	Beta	34.72	26.04	43.40
Live birth rate in 2nd FET cycle of GnRHa group	Beta	35.63	26.72	44.54
Live birth rate in 3rd FET cycle of GnRHa group	Beta	24.14	18.11	30.18
Live birth rate in 4th FET cycle of GnRHa group	Beta	18.18	13.64	22.73
Live birth rate in 1st FET cycle of CC cotreatment group	Beta	35.50	26.63	44.38
Live birth rate in 2nd FET cycle of CC cotreatment group	Beta	52.30	39.23	65.38
Live birth rate in 3rd FET cycle of CC cotreatment group	Beta	21.10	15.83	26.38
Live birth rate in 4th FET cycle of CC cotreatment group	Beta	50.00	37.50	62.50

Note: In the cost-effectiveness analysis, the probability of live birth rate was calculated as the average live birth over the embryo transfer cycles. We generated estimates of probabilities of live birth per embryo transfer. Only direct medical costs were included in the cost- effectiveness analysis of this study, as indirect costs are highly variable and difficult to calculate.

a Ultrasound monitoring, sex hormone testing, consultation, and drugs costs during ovulation induction are all included. b The cost of luteal support here is restricted to the cost of drugs administered 12-14 days following embryo transfer. c The cost of luteal support drugs for the first 8-10 weeks after embryo transfer is also included. CC cotreatment, clomiphene citrate and gonadotropin cotreatment; GnRHa, Gonadotropin-releasing hormone agonists; OS, ovarian stimulation; FET, frozen embryo transfer.
